# Migratory Preseptal Cellulitis Secondary to Rhinosinusitis Complicated by Left Frontal Subdural Empyema in a Child: A Case Report

**DOI:** 10.1155/crop/5117052

**Published:** 2026-04-06

**Authors:** Frank Sandi, Jacinta Feksi, Henry Humba, Suzana R. Lukoo

**Affiliations:** ^1^ Department of Ophthalmology and Vision Sciences, The University of Dodoma Medical School, Dodoma, Tanzania; ^2^ Department of Ophthalmology, Benjamin Mkapa Hospital, Dodoma, Tanzania; ^3^ Department of Neurosurgery, Benjamin Mkapa Hospital, Dodoma, Tanzania; ^4^ Department of Radiology and Imaging, Benjamin Mkapa Hospital, Dodoma, Tanzania

**Keywords:** migratory preseptal cellulitis, rhinosinusitis, subdural empyema

## Abstract

Preseptal cellulitis is a common pediatric infection involving the eyelids and surrounding periocular tissues. It is typically benign and rarely progresses to intracranial complications, unlike orbital cellulitis. However, when secondary to rhinosinusitis, the infection may behave unpredictably and extend beyond expected anatomical boundaries. We report a rare case of migratory preseptal cellulitis in a 12‐year‐old boy, initially presenting with unilateral eyelid swelling, erythema, and fever. Despite prompt initiation of empirical intravenous antibiotics, the condition failed to improve and instead demonstrated an unusual migratory pattern, spreading superiorly to the forehead and crossing the midline to involve the contralateral eye. This atypical progression raised concern for deeper infection and prompted advanced imaging. Computed tomography and magnetic resonance imaging revealed maxillary sinusitis with intracranial extension, resulting in a left frontal subdural empyema. The patient underwent multiple surgical interventions, including incision and drainage of the eyelid, craniotomy for empyema evacuation, and functional endoscopic sinus surgery to address the underlying sinonasal source. Broad‐spectrum intravenous antibiotics were continued, as there was no growth after 5 days of incubation. Following combined surgical and medical management, the patient made a full recovery within 20 days, with complete resolution of neurological and ocular symptoms. This favorable outcome underscores the importance of early recognition and aggressive treatment of intracranial complications arising from seemingly localized infections. This case highlights that intracranial extension can occur even in preseptal cellulitis, particularly when symptoms worsen or fail to respond to standard therapy. Migratory eyelid swelling should heighten clinical suspicion for atypical or progressive disease. Prompt imaging, identification of the primary infectious source, and timely multidisciplinary intervention are essential to prevent life‐threatening complications.

## 1. Background

Preseptal cellulitis is an infection in front of the orbital septum, a thin, fibrous membrane that is anatomically important in the evaluation of orbital infections and acts as a barrier between the eyelids and the orbit [1, 2]. Infections in the posterior part are classified as orbital cellulitis [3]. Rhinosinusitis can complicate onto orbital cellulitis, which can easily spread to the brain unlike preseptal cellulitis due to the presence of the orbital septum [4]. Our patient had no anatomical particularity that disposed him to preseptal cellulitis.

There is usually no need for radiological examination such as imaging with magnetic resonance imaging (MRI) or CT unless suspected orbital cellulitis, eyelid abscess is present, or there is a failure to respond to therapy [5]. Antibiotic therapy should be initiated as soon as possible [6].

We present a rare pediatric case of migratory preseptal cellulitis with radiologically confirmed maxillary sinusitis and left frontal subdural empyema, which required combined eyelid drainage, craniotomy, and endoscopic sinus surgery [7]. This case highlights diagnostic pitfalls, the role of imaging in atypical presentations, and the importance of early, coordinated surgical and medical management to achieve a favorable outcome [3, 8, 9].

## 2. Case Presentation

A 13‐year‐old boy was brought to the hospital′s emergency department with the complaints of fever, loss of appetite, and swelling in the right eye more than the left eye, which was associated with confusion‐talking irrelevant words. His eye swelling had started 3 days before coming to the hospital and was followed with fever and headache a day before hospital attendance. He denied history of trauma or similar episodes previously.

On physical examination, his general condition was good; he was conscious with Glasgow Coma Scale of 14/15, and his axillary body temperature was 39.4°C. Blood pressure of 125/75 MmHg, heart rate of 98 b/m, respiratory rate of 14 b/m and 100% oxygen saturation on room air. An MRI was ordered, and an ophthalmologist review was sought.

On ocular examination, there was marked edema in the right upper eyelid and slight lid edema of the left eye; his visual acuity was 6/6 in both eyes with no conjunctival hyperemia nor chemosis. IOP was 13 RE and 14 LE, anterior segment examination was normal, lens clear, and posterior examination was normal with CDR of 0.2 in both eyes. His eye movements were painless and normal in all directions. There was good Bell′s phenomenon in both eyes. System examinations were normal; there were no signs of meningeal irritation.

Laboratory findings, white blood cell (WBC) count was slightly higher 15.95 × 10^3^/*μ*L, neutrophilia of 84.2% was also evident, absolute lymphocytopenia of 0.96 × 10^3^/*μ*L; the rest of blood parameters were normal. Blood electrolytes, liver, and kidney function tests were normal. The patient was admitted in surgical ward with a diagnosis of preseptal cellulitis and meningitis. Medical management was initiated with intravenous ceftriaxone, oral amoxicillin with clavulanic acid, and oral paracetamol tablets. Blood for culture and sensitivity (aerobic/anaerobic) was taken and there was no bacteria growth after 5 days of incubation.

Contrast‐enhanced MRI of the brain performed on the third day of admission demonstrated extensive inflammatory changes involving the paranasal sinuses consistent with pansinusitis, including bilateral maxillary sinus opacification with mucosal thickening of the ethmoid, sphenoid, and left frontal sinuses, whereas the right frontal sinus was nonpneumatized (Figures 1a, 1b, and 1c). There was diffuse soft‐tissue swelling and postcontrast enhancement of the left upper eyelid and preseptal tissues consistent with preseptal cellulitis, with a normal contralateral orbit (Figure 1d). Additionally, a crescentic extra‐axial collection along the left frontal convexity showing postcontrast enhancement was identified, consistent with a left frontal subdural empyema, with otherwise normal brain parenchyma (Figure 1e).

Figure 1MRI images showing (a) T2‐weighted coronal view demonstrating bilateral maxillary sinusitis; (b) T2‐weighted axial view demonstrating ethmoid and sphenoid sinusitis; and (c) T2‐weighted axial view demonstrating left frontal sinusitis with a nonpneumatized right frontal sinus. Postcontrast T1‐weighted MRI images showing (d) axial view demonstrating left preseptal cellulitis with a normal right orbit, and (e) sagittal view demonstrating left frontal subdural empyema with otherwise normal brain parenchyma. (f) Right eye preseptal abscess (red circle). (g) Right eye healing (a scar after draining‐green arrow) and left eye starting swelling (red circle). (h) Brain CT scan showing subdural empyema on the left frontal‐parietal lobes (red arrows). (i) Patient recovered and no more eyelids swelling.

**Figure:**
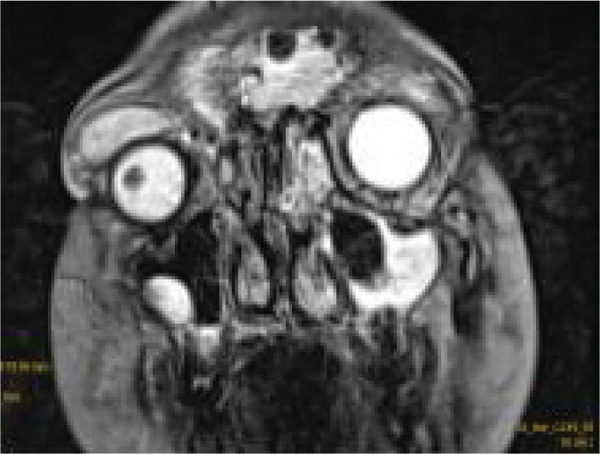
(a)

**Figure:**
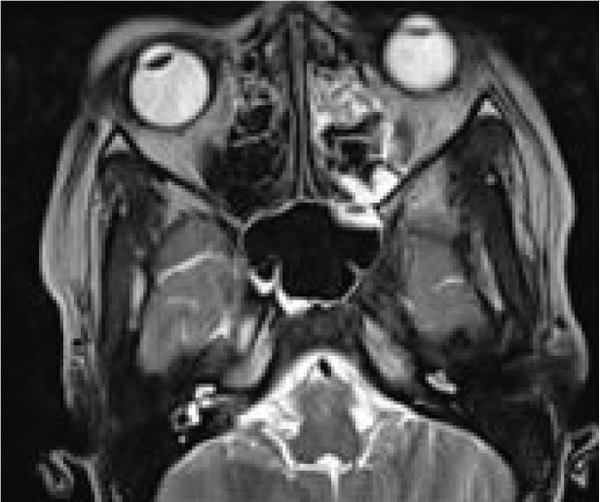
(b)

**Figure:**
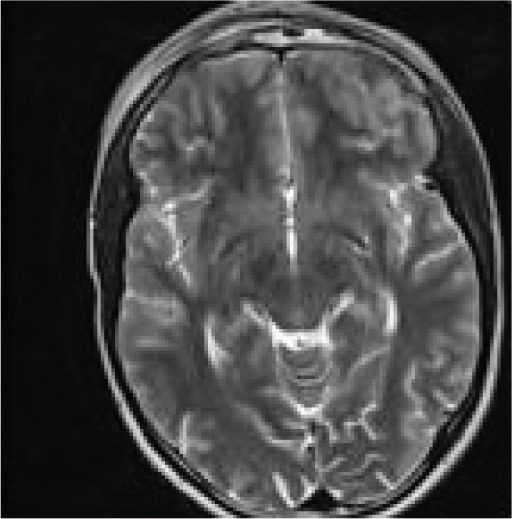
(c)

**Figure:**
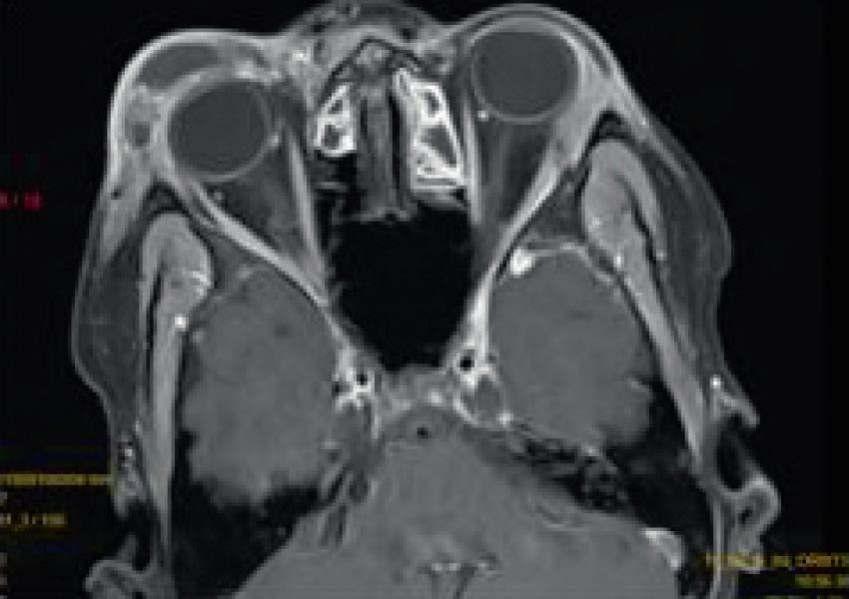
(d)

**Figure:**
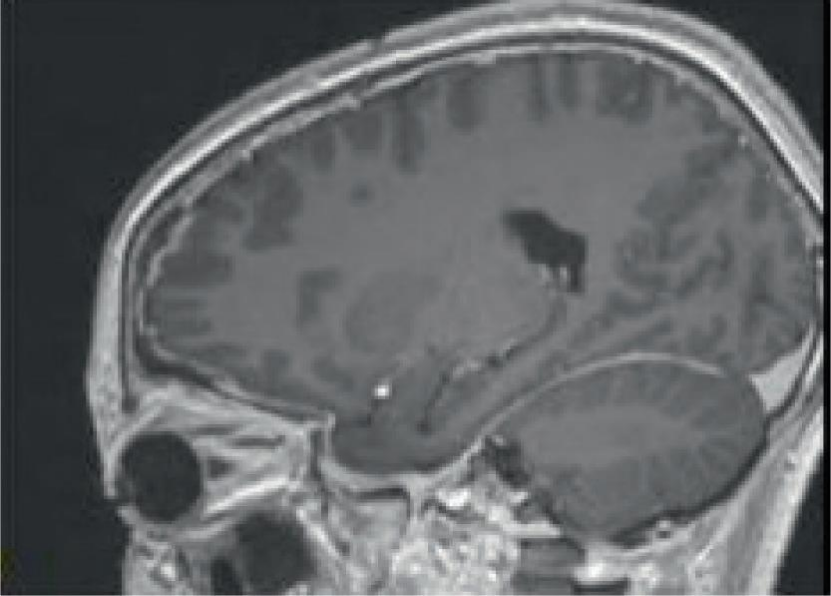
(e)

**Figure:**
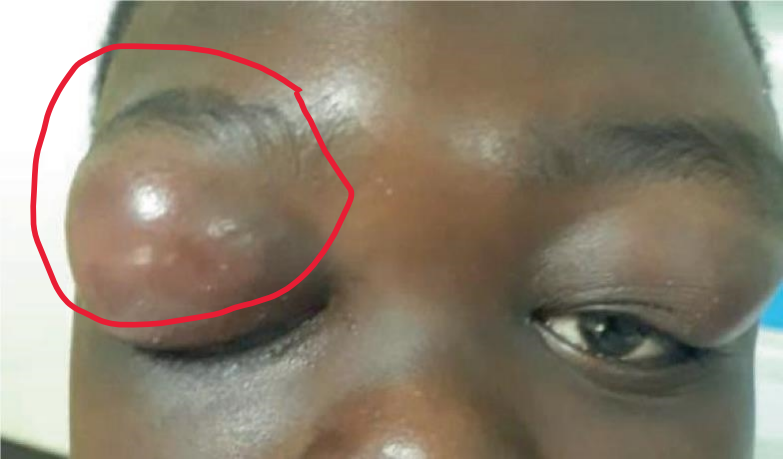
(f)

**Figure:**
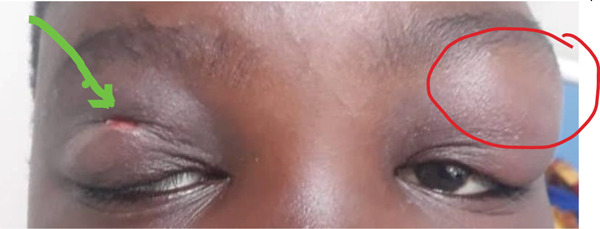
(g)

**Figure:**
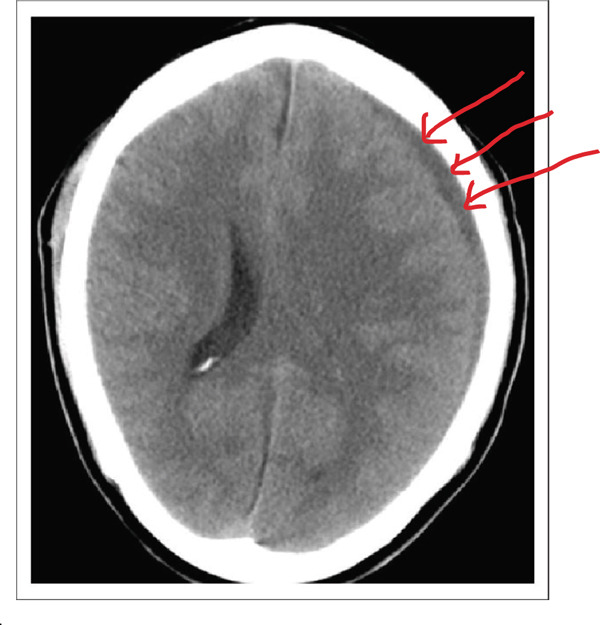
(h)

**Figure:**
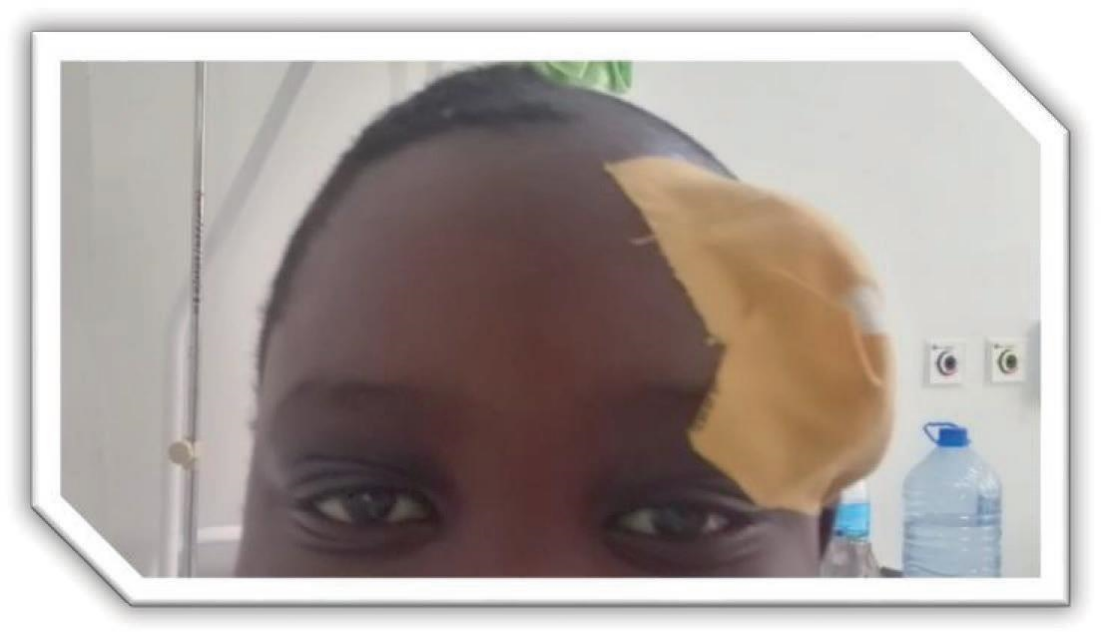
(i)

Day 5 in the ward and the right upper eyelid kept on swelling with spikes of fever up to 40°C despite treatment. ENT department was consulted due to increased swelling in the left eye despite treatment. The working diagnosis was by now preseptal cellulitis and rhinosinusitis involving ethmoid and maxillary sinus. ENT specialists advised the patient to continue with the mentioned antibiotics and follow up for any positive response.

Day 6 in the ward, examination of the right upper eyelid had signs of preseptal abscess (Figure 1f), which was drained under general anesthesia and the pus taken for culture and sensitivity. The patient′s fever stabilized and IV metronidazole 400 mg tds was given in addition to IV ceftriaxone given initially. There was no bacterial growth from the pus taken; hence a regrowth was done again.

Day 7 and 8, the patient was vitally stable, no fever, good appetite, and the patient was still under the mentioned antibiotics, and the team was happy as they saw the boy recovering and about to be discharged.

Day 9 in the ward, the right eye swelling subsided and the left upper eyelid started swelling too. There were no signs of orbital cellulitis. The fever spikes started again and there were signs of eyelid abscess with no eyelid tenderness (Figure 1g). The abscess was again drained under general anesthesia where approximately 30 mL of pus, greenish yellowish in color was drained. Puss for culture and sensitivity was taken again. Ceftriaxone was stopped and the patient was started on IV amoxiclav 625 mg tds in combination with IV metronidazole 400 mg tds and paracetamol 1 g tds tablets.

Day 10 in the ward, the patient started convulsing with fever strike, confusion, loss of memory, and aphasia. Emergency CT scan was done where he was found to have subdural empyema (Figure 1h). The neurosurgery team was consulted, and emergency draining of the empyema was done on the same day.

Day 11 in the ward, the patient′s memory was back but had slurred speech, no fever, and upper eyelid swelling has subsided (Figure 1i). Our patient was smiling and eyelids′ swellings had gone but the team had not known that they still have the real culprit to deal with, and this was rhinosinusitis, which was seen on the initial MRI (Figures 1a, 1b, and 1c).

ENT team had advised letting the patient stabilize before the team goes in to do sinus washouts, and yes, 1 week after, the patient was taken back to theater. Sinus washout was done under functional endoscopic sinus surgery (FESS) procedure.

The patient′s treatment was completed in 4 weeks and discharged home with full recovery as narrated in Table 1. The patient is currently going on with his studies.

**Table 1:** Timeline illustrating the clinical progression of the case.

Timeframe	Clinical events
Day 0–2	Onset of acute swelling of RE, with nasal congestion, facial pressure, and low‐grade fever associated with confusion
Day 3	Attended emergency department at our hospital and had started confusions and RE swelling progressing
Day 4–9	Admitted in the ward and on treatment and Investigations were done. The RE upper eyelid continued to swell and ENT consulted
Day 10–12	Emergence of neurological symptoms (persistent headache, vomiting, and irritability/photophobia) and concern for intracranial extension was suspected
Day 13‐14	Admitted, left eye started to swell, fever spikes again, and CT reveals subdural empyema. Neurosurgery team was consulted
Day 15–21	Continuation of IV broad‐spectrum antibiotics, ENT and neurosurgery consultations, and surgical drainage and memory regained
Week 4	Clinical improvement: resolution of fever and eyelid swelling, neurological symptoms improve, and imaging shows reduction in abscess size. ENT team went in for FESS and the patient was discharged after good recovery

## 3. Discussion

Rhinosinusitis is a common condition with a prevalence of 6–12 in 100 children, and most cases never reach a stage for admission but are managed medically as outpatient cases as they rarely lead to complications. Majority of rhinosinusitis arise from viral and later can proceed to secondary bacterial infection with various complications manifestation [9, 10].

Complications of rhinosinusitis in childhood include preseptal cellulitis, orbital cellulitis, and intracranial complications such as meningitis and cavernous sinus thrombosis [11, 12]. Among these, orbital cellulitis is the commonest, with intracranial complications being the least common. Orbital complications include orbital cellulitis, orbital subperiosteal abscess, edema in orbital muscles, inflammation, and decreased vision. Intracranial complications include epidural empyema, subdural empyema, intracerebral abscess, meningitis, Pott′s puffy tumor, and cavernous sinus thrombosis [4, 13, 14].

Frequent occurrence of ocular complications in rhinosinusitis is due to the anatomical neighborhood with frontal sinus at the top, the ethmoidal sinus medially, and the maxillary sinus at the bottom. The paper‐thin lamina papyracea, located medially, may cause an infection in the ethmoid sinus to easily spread to the orbit.

Orbital complications of rhinosinusitis were first classified by Chandler et al. and have been revised recently [15, 16]. Group 1 (preseptal cellulitis) infection affects only the eyelid, Group 2 (orbital cellulitis) is the diffuse edema in orbital structures, Group 3 is the subperiosteal abscess, Group 4 is the abscess development in orbital structures, and Group 5 is the infection reaching cavernous sinuses and resulting in cavernous sinus thrombosis [2, 17]. Our study case with preseptal cellulitis and subdural empyema as a complication of rhinosinusitis is meant to draw attention to the intracranial complications of rhinosinusitis.

There are factors that help differentiate between preseptal cellulitis and orbital cellulitis. In preseptal cellulitis, the vision is usually spared with no afferent pupillary defect nor proptosis [3]. Extraocular movements are usually normal and painless. That said, our case had fever and preseptal cellulitis due to rhinosinusitis. There were no observed signs of orbital cellulitis, both clinically and in the MRI, which are usually hyperintense areas around the globe and behind it, causing proptosis and not ptosis [18].

There were also reported signs of confusion, headache, convulsion, and aphasia that would suggest a central nervous system infection clinically [14, 19]. The diagnosis of epidural empyema was confirmed upon detection of radiological dural contrast enhancement in MRI [20]. The reason why no growth was detected in blood culture might be related to the intravenous antibiotic use before the FBP investigation. In our case, subdural empyema was determined to be caused by the intracranial spread of rhinosinusitis involving the ethmoid and maxillary sinus. So, in this case, probably we were dealing with two separate diseases, as rarely can rhinosinusitis result in preseptal cellulitis. Hence, the uniqueness of this case we are reporting.

Several studies have shown intracranial and intraorbital extension of rhinosinusitis as a complication of rhinosinusitis. In all studies, intraorbital extension is more common with a range of 60%–80%, whereas intracranial extension ranges from 20% to 40% and both extensions have been observed in 11% of cases [11, 14, 19, 21]. In the majority of cases, the intraorbital extension is usually orbital cellulitis, but in our case, it was preseptal cellulitis which had a migratory nature from right eye to left eye and then to the brain. This migratory preseptal cellulitis has not been reported anywhere throughout the literature we searched.

In most cases reports and studies done, the intracranial spread pattern of 60% of patients has been shown to be dural thickening in 20% of cases, an epidural abscess in 15% of patients, subdural empyema in 15% of patients, frontal bone osteomyelitis in 9%, subdural empyema in 4%, and sinus thrombosis in 1% [3, 5, 22]. This is in line with our patient who had subdural empyema, which happens in some groups of patients, especially teenagers, in which our patient was in that group. Studies have shown that 65% of patients with intracranial spread also had orbital complications of sinusitis, most commonly orbital cellulitis, which was detected in 45% [9]. Unlike in our case, this was preseptal cellulitis, which is a rare finding to spread as a subdural empyema.

In Schlemmer et al.′s retrospective study, distribution of intracranial complications was reported as 72% subdural collection, 11% extradural collection, 6% subdural + extradural combined collection, 6% edema/cerebritis, and 5% intracerebral abscess. In this study, they stated that there was a high mortality rate of 20.7% in patients with intracranial complications [11, 23]. Complicated sinusitis was still very common in emerging countries, and that young adolescent males were at the highest risk [11]. In another meta‐analysis study of intracranial complications of rhinosinusitis in childhood by Patel et al., 180 patients in 16 studies were evaluated and showed that the most common intracranial complications were subdural empyema (49%), epidural abscess (36%), cerebral abscess (21%), and meningitis (10%) [24].

Zhao et al. examined the complications of acute rhinosinusitis in pediatric patients. The results showed that, out of 724 patients with acute rhinosinusitis, 64 patients were hospitalized due to complications, out of which, 29.7% were due to intracranial complications (epidural/subdural abscess and cavernous sinus thrombosis) [25]. Our client was a male young teenager. Lucky enough, due to close follow up and management, he survived and did not get any residual complication. Imaging was checked for CST signs and there was no evidence of thrombosis seen in the cavernous, which ruled out possibilities of this fatal complication [26].

In another retrospective study, 104 pediatric patients were hospitalized with the complication of acute rhinosinusitis. The findings showed that the rate of hospitalization of the male gender was significantly higher. The age of distribution of patients with only orbital complications was small, compared with those with intracranial complications (mean: 6.5/12.3 years) [27, 28]. This is in line with our case who was a male aged 13 and had preseptal cellulitis and intracranial involvement. This shows there is a lower rate of orbital involvement at an advanced age.

Another differential diagnosis would have been rhino‐orbita–cerebral mucormycosis, which has been described its histopathology well by Jeican et al. where they found out that it is currently associated with COVID‐19 and hence coined a new clinical entity called COVID‐19–associated rhino‐orbital mucormycosis (CAM), which has been popular during the second wave of COVID‐19 especially in Asia [29, 30]. Mortality in this condition is usually high and has been reported to range from 30% to 70% and it is a fatal rapidly progression disease if delay in diagnosis. [31, 32]

MRI is the preferred method for detecting orbital soft tissue complications and intracranial complications, together with MR venography when cavernous sinus thrombosis is considered [16]. CT is the most widely accepted examination in the diagnosis of rhinosinusitis and shows the presence of complications such as orbital abscess and subperiosteal abscess. In our case [12], CT scan of the orbit and brain and MRI were performed, and signs of rhinosinusitis () and preseptal cellulitis were detected, and blood culture and sensitivity were performed, which helped in the early diagnosis and timely management.

Lumbar puncture for CSF analysis was not done in our case as it has been shown to be contraindicated due to the risk of brain herniation as it has been agreed in Consensus guidelines for lumbar puncture in patients with neurological diseases [33]. Hence, the clinical examination and the CT scan were enough to make a diagnosis of subdural empyema in this case.

In conclusion, rhinosinusitis, which is common in childhood, maintains its importance due to its complications and mortality if not properly managed. This case demonstrates that preseptal cellulitis can, in rare circumstances, herald intracranial spread, particularly when associated with rhinosinusitis or when clinical features are atypical or progressive. Migratory eyelid swelling, lack of response to appropriate empirical therapy, or extension across anatomical boundaries should prompt early cross‐sectional imaging to exclude deeper infection. Care should be taken after the orbital swelling has subsided as new symptoms of intracranial extension can become evident even after the orbital signs have gone like in our case.

**Nomenclature**
b/m: beats/minutes
C: centigrade
CAM: COVID‐19‐associated rhino‐orbital mucormycosis
CDR: cup disc ratio
COVID‐19: corona virus disease 19
CSF: cerebral spinal fluid
CST: cavernous sinus thrombosis
ENT: ear, nose, and throat
FESS: functional endoscopic sinus surgery
IOP: intraoperative pressure
IV: intravenous
LE: left eye
MmHg: millimeters of mercury
MRI: magnetic resonance imaging
PO: postal office
RE: right eye
Tds: three times a day
WBC: white blood cells

## Funding

No funding was received for this manuscript.

## Ethics Statement

The publication of this case report together with the data collection and management involved was in accordance with the guidelines of the Institutional Review Board and Ethics Committee of our institution. The need for approval was waived, but the consent to participate was nonetheless taken from the patient′s parent.

## Consent

Written consent was obtained for the publication of the case report together with the patient′s photographs from the patient′s parent.

## Conflicts of Interest

The authors declare no conflicts of interest.

## General Statement

*Case Limitation*. This case report has several limitations. First, the unusual progression from rhinosinusitis to shifting eyelid involvement may represent an atypical presentation rather than a reproducible clinical pattern. Second, management decisions—including timing of imaging and choice of antibiotics—were influenced by resource availability, which may differ across settings and affect reproducibility. Third, Rhino‐orbital mucormycosis would have been considered as a differential diagnosis here but it was not as it has been coined as a new clinical entity. Finally, tissue samples collected during functional endoscopic sinus surgery were not studied through histopathological examination, scanning electron microscopy, and transmission electron microscopy, which would have yielded more insight onto this case.

Despite these limitations, the case provides valuable insight into an uncommon but clinically significant progression from sinusitis to preseptal cellulitis and ultimately subdural empyema, underscoring the importance of early recognition of red‐flag symptoms like the migratory nature revealed in this case.
